# Computer‐Based Design to Improve *Bacillus thuringiensis* Chitinase for Industrial Applications

**DOI:** 10.1002/open.70190

**Published:** 2026-04-01

**Authors:** S. G. Sree Agash, G. Chandrasekhar, A. S. Vinutha, S. Akshay, P. Kanishk, R. Rajasekaran

**Affiliations:** ^1^ Quantitative Biology Lab, Department of Integrative Biology, School of Bio Sciences and Technology Vellore Institute of Technology (VIT Deemed to be University) Vellore India

**Keywords:** chitin, chitinase, nucleophilicity, reaction coordinates, site‐directed mutagenesis

## Abstract

Chitin serves as a vital biopolymer across multiple industrial biotechnology sectors, and its extraction from seashell waste through enzymatic bioconversion offers an efficient and environmentally sustainable approach. Chitinase plays a key role in this process; however, strategies to enhance its catalytic performance remain limited. In this study, in silico site‐directed mutagenesis was employed to improve the enzyme's activity while preserving its structural stability. Mutation sites were identified within the catalytic domain based on high mutability scores and functional relevance. Six hotspot mutations V215A, V215F, S262G, R264H, F288L, and G291A were selected for further evaluation. Interaction analyses revealed that the beneficial mutants demonstrated stronger substrate affinity compared to the native enzyme. Furthermore, molecular orbital analysis indicated that substrate binding to these mutants, particularly the G291A variant, exhibited enhanced nucleophilic and electrophilic characteristics. Notably, the G291A mutant showed reduced activation energy of 49.53 kcal/mol, in contrast to 97 kcal/mol for the native enzyme, signifying improved catalytic efficiency. Overall, this study highlights the potential of computationally guided site‐directed mutagenesis as a rational approach for optimizing chitinase activity and advancing enzyme engineering for bioconversion applications.

## Graphical abstract

Hotspot‐guided mutagenesis identifies Gly291 as a key catalytic determinant in *Bacillus* *thuringiensis* chitinase. Substitution with alanine improves substrate accommodation and lowers the activation barrier by ∼47%, reducing it from 94.66 to 49.83 kcal/mol. Structural integrity is retained while catalytic efficiency is enhanced, demonstrating a rational strategy for engineering high‐performance chitinases.

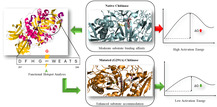

**Abbreviations**
QM, Semiempirical quantum mechanics
SMD, Steered molecular dynamics
HOMO, Highest occupied molecular orbital
LUMO, Lowest unoccupied molecular orbital
R, Reactant energy
TS, Transition state energy
P, Product energy

## 1 Introduction

Chitin serves as a major structural component in the exoskeletons of arthropods, forming an integral part of their respiratory linings. It is abundantly distributed among marine organisms, especially within the shells of crustaceans such as shrimp and lobsters [1]. After industrial processing, these shells are typically discarded as chitinous waste, which can pose environmental hazards, by disrupting the pH balance of surrounding water and soil systems [2]. Approximately 30% of chitinous waste consists of chitin molecules, a valuable raw material employed in the production of various valuable biopolymers, such as hydrogels, edible films, and chitin oligosaccharides. These biopolymers find wide‐ranging applications across biomedical, agricultural, and food processing industries [3]. Various approaches have been developed to extract or process chitin from chitinous waste, among which green extraction via enzyme degradation is recognized as one of the most efficient methods, enabling high‐yield chitin production in biotechnological industries [4]. This enzymatic degradation is catalyzed by chitinase, a hydrolytic enzyme belonging to the glycoside hydrolase families GH18 and GH19 [5].

Chitinase functions by cleaving the beta‐1,4 glycosidic linkages within chitin molecules, thereby shortening the polymer chains and converting them into chitin oligosaccharides [6]. This enzymatic action not only facilitates biodegradation but also aids in waste reduction, thereby positioning chitinases as key contributors across various biotechnological sectors [7]. Chitinases belonging to the GH18 family are the most predominant enzymes utilized in bioprocessing due to their ubiquitous presence and widespread distribution across diverse species [8]. These GH18 chitinases are characterised by an (α/β)_8_‐TIM barrel catalytic cleft, which accommodates multiple binding domains, thereby enhancing their chitin binding affinity [9]. In contrast, GH19 chitinases primarily found in plants, exhibit limited substrate specificity, resulting in comparatively lower catalytic activity [10]. Among GH18 members, bacterial chitinases are particularly favoured and extensively studied because of their high expression stability and substrate specificity [11, 12, 13, 14]. Notably, chitinases derived from *Bacillus* species are recognized for their superior thermal tolerance and remarkable efficiency in chitin degradation [7, 15, 16, 17, 18].

Furthermore, the Carbohydrate‐Active Enzymes database documents multiple *Bacillus* species strains, among which *Bacillus thuringiensis* is notably well‐studied [19, 20] . This species plays a pivotal role in agriculture through its ability to degrade chitin, thereby contributing to the development of biopesticides [21]. *B. thuringiensis* is particularly renowned for its dual functionality exhibiting both chitin‐degrading and insecticidal properties, which makes it highly effective against a broad range of pests. However, despite its agricultural and industrial applications, computational investigations focusing on site‐directed mutagenesis and its influence on catalytic efficiency remain limited and underexplored [22, 23, 24]. Although previous studies have explored the effects of mutations on the thermostability and enzyme–substrate interactions of chitinase, a comprehensive molecular modelling analysis detailing the conformational dynamics of the enzyme–substrate reaction is still lacking. Such an analysis is essential to deepen the understanding of chitinase site‐directed mutagenesis and to optimize its catalytic efficiency for industrial applications [25, 26].

Accordingly, in the present study, in silico site‐directed mutagenesis was carried out on substrate‐specific binding residues located near the key catalytic residue, glutamate at position 211 (E211), in *B. thuringiensis* chitinase. To elucidate the influence of adaptive mutations on the catalytic domain, a robust reaction modelling approach based on semiempirical quantum mechanics (QM) was employed. This QM‐based analysis enables atomic‐level evaluation of enzyme–substrate interactions governing the bond cleavage mechanism during chitin hydrolysis. Furthermore, it provides valuable insights into how beneficial mutations enhance the chitinolytic activity of *B. thuringiensis* chitinase through optimized catalytic reside‐substrate interactions.

## 2 Methodology

### 2.1 Three‐Dimensional Structural Refinement

The 3D crystal structure of the *B. thuringiensis* chitinase enzyme (PDB ID: 6BT9) was retrieved from the Protein Data Bank [27, 28]. The retrieved structure was then subjected to energy minimisation using the steepest descent algorithm in YASARA to achieve geometric optimisation [29]. For the substrate, the chitin molecule N‐acetyl‐beta‐D‐glucosaminyl‐(1‐>4)‐N‐acetyl‐beta‐D‐glucosamine (chitobiose) (PubChem CID: 439 326) was selected for bioconversion analysis [30]. Further, the substrate structure was geometrically optimized using the semiempirical MOPAC module integrated within the YASARA molecular modelling suite [29].

### 2.2 Hotspot Mutational Analysis

Subsequently, HotSpot Wizard 3 was utilised to identify adaptive site‐targeted functional hotspot mutations within the chitin‐binding domain. This tool employs an automated mutational design framework that integrates in‐house libraries and computational modules to assess protein functionality and stability following amino acid substitutions. It further incorporates both structural and evolutionary data analyses to compute and validate functional hotspots. In this study, the catalytic residue E211 was preserved during the in silico site‐directed mutagenesis, as its selection facilitates accurate determination of binding pockets and tunnel regions. All remaining residues were analysed to calculate the accessible surface area, enabling differentiation between buried residues and exposed residues. This classification was based on Kabsch & Sander's implementation of the Lee and Richards rolling‐probe algorithm, thereby aiding in the identification of potential hotspot residues [31].

Based on residue location and associated parameters, the identified mutations were categorised into two groups: functional and stability‐related. The computational results were evaluated across four distinct protein engineering strategies: identification of functional hotspots within protein structures, assessment of structural flexibility, sequence consensus analysis, and examination of correlated structural hotspots. Among these, the functional hotspot protein engineering strategy was selected for detailed analysis.

Next, specific criteria were applied to filter the hotspot residues in proximity to the catalytic residue. The selected residues were required to meet the following conditions: 1) a high mutability score (>=6), indicating their position within a highly mutable region; 2) exclusion from critical functional roles, such as catalytic residues, 3) localization within the access tunnel location, and 4) positioning within the catalytic site pocket.

These criteria ensured that the identified residues were more likely to enhance the functional efficiency of the key catalytic residue without compromising the enzyme's structural stability.

The selected hotspot residues were subsequently mutated according to the specifications detailed in the residue details section. The mutational landscape wizard was employed to determine the percentage probability of each mutation preserving enzymatic function, thereby identifying residues with the highest potential for beneficial mutability at specific positions. The beneficial mutations were then individually introduced into the enzyme structure to enable a comprehensive comparative functional analysis between each mutant variant and the native *B. thuringiensis* chitinase. The resulting mutant enzyme structures were further optimized for subsequent analyses.

### 2.3 Thermal Denaturation Analysis

Subsequently, simulated structural denaturation was performed to evaluate the structural stability and flexibility of both the native chitinase and its mutant variants using ProFlex software. This tool provides detailed conformational insights by distinguishing rigid and flexible regions within the protein structure through hydrogen bond dilution analysis. In this process, the structure was systematically denatured under controlled conditions, wherein hydrogen bonds were progressively removed from the weakest to the strongest, and the corresponding energy changes were recorded. The ProFlex platform analysed the flexibility region of enzyme structures using a 3D constraints counting algorithm based on rigidity theory [32, 33].

### 2.4 Molecular Docking

Besides, the molecular interactions of the enzyme structures and the chitin molecule were computed using the Autodock 1.5.6 docking software, which employs a search algorithm and scoring function to assess the probable binding sites and conformations in the enzyme–substrate complex. For receptor (enzyme) preparation, nonpolar hydrogens were added to the enzyme structure, followed by the assignment of Kollman charges. Subsequently, Gasteiger charges were calculated to accurately model the electrostatic interactions among atoms. Thereafter, torsional parameters were defined for the ligand molecule to specify its rotational bonds. Subsequently, the receptor molecule was kept in a rigid state, and the active site of the enzyme structures (E211) was designated. The grid box dimensions were set to 40 × 40 × 40 Å, with the *x*, *y*, *z* coordinates aligned to encompass the positions of the active site residues. This, in turn, allows the calculation of interaction energy grids around the receptor's active site to generate receptor grid points. Further, to generate ligand binding conformation in the active site, Lamarckian genetic algorithm was used. For the computation of the binding free energy, AutoDock utilises a semiempirical free energy force field. Subsequently, the best docking conformation and the binding affinity scores were investigated based on the ranking‐oriented clustering of the most efficient substrate–enzyme conformations [34].

### 2.5 Steered Molecular Dynamics

The dissociation behaviour of the substrate molecule from various enzyme structures offers valuable insights into substrate accommodation within the binding pocket and the affinity of surrounding residues for substrate binding. This evaluation was performed using steered molecular dynamics (SMD) simulations, which further facilitate a detailed understanding of how beneficial mutations dynamically influence the enzyme's conformational behaviour and its interaction with the substrate molecule. Here, a constant pulling force was applied systematically, during which the molecular response of the simulated system was analysed. The pulling force was set at 2000 (kJ/mol) for the system, and the dissociation distance was set to 30 Å for the substrate molecule to separate from the enzyme. Finally, the time taken to completely dissociate the substrate from the chitinase and its mutant enzyme structures was measured, thus illustrating the binding interaction between the substrate and enzyme structures [35, 36].

### 2.6 Molecular Orbital Analysis

The highest occupied molecular orbital (HOMO) and lowest unoccupied molecular orbital (LUMO) energy separation defines the chemical reactivity and kinetic stability of the substrate molecule [37]. Accordingly, the molecular orbital energies of the substrate bound to the enzyme structures were computed quantum semiempirically. These molecular orbital energy differences were ascertained using the AMPAC‐11 molecular modelling tool, in which the semiempirical Austin Model‐1 (AM‐1) method was used for geometry optimisation and calculation of HOMO–LUMO energy separation values (ΔE) [38].

### 2.7 Reaction Modelling Analysis

To investigate the enzyme kinetics of chitinase and its beneficial mutants, the enzyme–substrate reaction pathway was analysed using TRITON, a semiempirical quantum mechanical simulation software. Here, MOPAC's core semiempirical engine, used for structural optimisation and energy calculations, computes the reactant energy (R), transition state energy (TS), and product energy (P). When integrated with the DRIVER module, the system generates reaction coordinates through atomic coordinate scans. These coordinates serve as the initial reference points for defining the reaction pathway of the enzyme–substrate complex. Specifically, two key atomic coordinates were considered: the oxygen atom of the catalytic residue (E211) and the C4 atom of the substrate molecule involved in the glycosidic bond interaction. These reaction coordinates facilitate tracking the bond formation and cleavage events occurring between the specified atoms [39].

## 3 Results and Discussion

### 3.1 Site‐Directed Mutational Analysis

**FIGURE 1:**
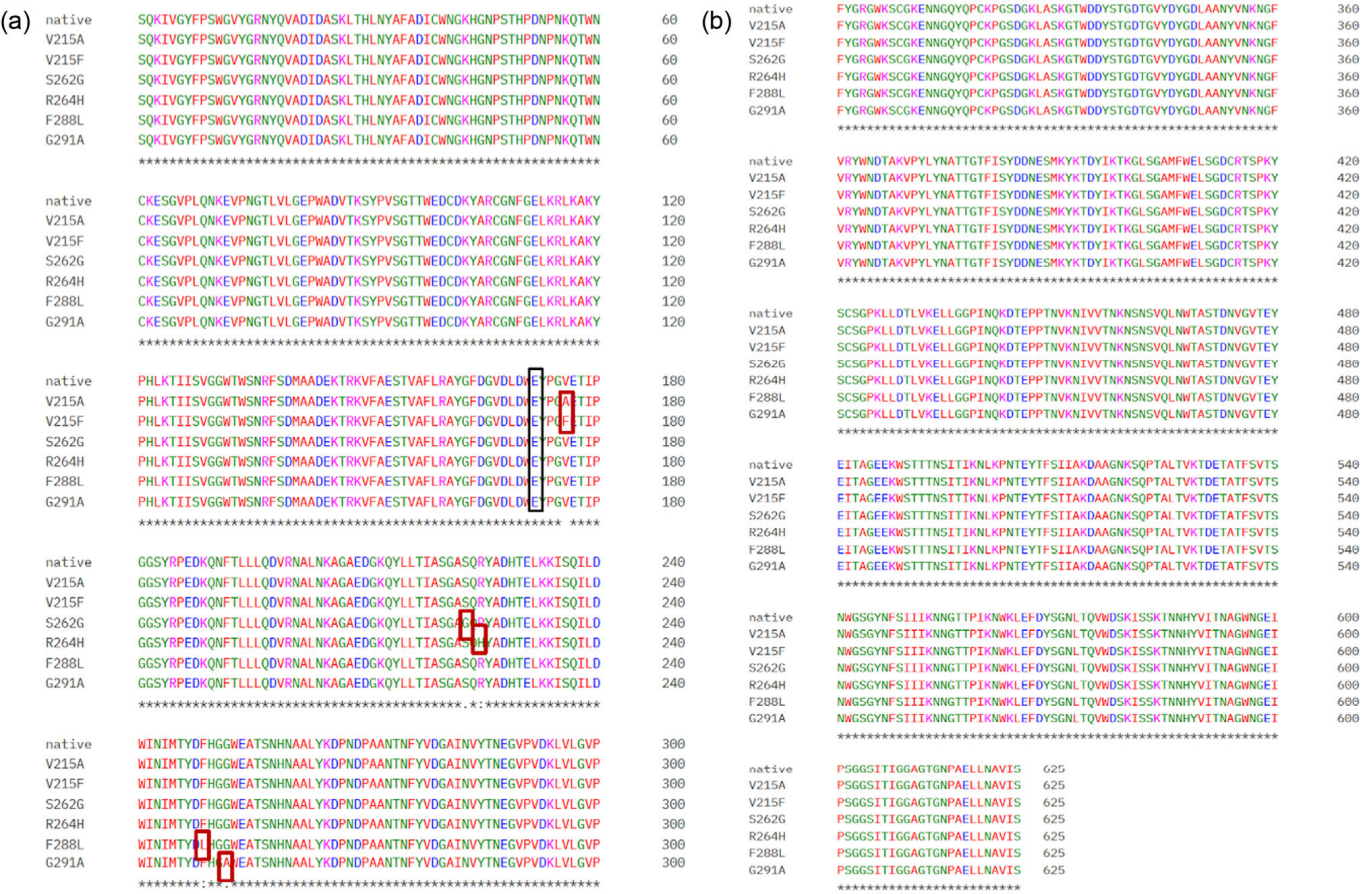
(a) and (b) The Multiple Sequence Alignment generated using Clustal Omega for the native chitinase and the functional hotspot mutants (V215A, V215F, S262G, R264H, F288L, and G291A). The mutated residues are highlighted within red boxes, indicating their respective substitution positions. The catalytic residue (E211), which remains conserved across all sequences, is highlighted in a black box.

The site‐directed mutational analysis was strategically designed to enhance the catalytic efficiency of *B. thuringiensis* chitinase while preserving its overall structural stability. Table 1 presents a systematic comparison of previously reported validation studies in which site‐directed mutations and structure‐guided modifications led to enhanced catalytic efficiency and functional performance. It also outlines the rationale adopted in this study, referencing established strategies such as binding‐groove engineering, stability enhancement, and active‐site modulation. Based on these insights, in silico mutagenesis (Table 2) was carried out to identify the most favourable amino acid substitutions. The mutability score of enzyme structure's residues, ranging from 6 to 9, is highlighted as highly mutable, as they serve as functional hotspots that are ideal targets for mutagenesis. The selected mutational positions between grades 6 and 9 are V215, S262, R264, F288, and G291, which are also positioned in close proximity to the key catalytic residue (E211) within the enzyme's active site pocket. Additionally, 100% preservation of the enzyme's functionality was observed for point mutations at V215A, S264G, and G291A. Whereas in positions S262G and F288L, the percentage of conservation of the enzyme's functionality decreased to 88. These interpretations further suggested that targeted substitutions are advantageous for maintaining the enzyme's structural conformation. Accordingly, these six beneficial mutations at the specified site, viz., V215A, V215F, S262G, R264H, F288L, and G291A, were considered for site‐directed mutational analysis, since they were predicted to enhance the activity of the enzyme without leveraging the enzyme's conformational framework. These mutants were utilised for further study. The Multiple Sequence Alignment generated using Clustal Omega for the native chitinase and all functional hotspot mutants (Figure 1) revealed that sequence variations are restricted to the targeted residue positions, while the catalytic residues and key structural motifs remain fully conserved. This conservation indicated that the engineered mutations are unlikely to compromise the enzyme's structural integrity.

**TABLE 1:** A systematic comparison of the previously reported validation studies where site‐directed mutations and structure‐guided modifications resulted in improved catalytic favourability and functional performance.

Enzyme source	Mutation site	Mutagenesis strategy	Evidence of enhancement	Key findings reported	Research source
*Serratia marcescens* ChiB	Glycine to Alanine, loop‐stabilising substitutions	Stability‐oriented modification strategy	Thermal denaturation and enzymatic activity retention assays	Enhanced thermostability with retained catalytic activity	[40]
*Serratia marcescens* ChiB	Active‐site prone mutation	Crystal‐structure targeted mutagenesis	X‐ray crystallography, inhibitor binding analysis	Local active‐site rearrangement affecting the catalytic environment	[41]
*Vibrio harveyi* GH18 chitinase	Substrate‐binding groove residues	Structural analysis of the enzyme–substrate complex	X‐ray crystallography structures with chitooligosaccharides	Identification of the positioning of multiple subsites of the substrate	[42]
*Paenibacillus* sp. chitinase	Rationale‐based selection of multiple mutations (S244C‐I319C/T259P)	Semirational design	Enzymatically active assays, thermal stability analysis	Improved catalytic performance and thermostability	[43]

**TABLE 2:** The mutational hotspot predicted sites and the percentage of their preservation of function.

Selected mutational positions	Mutability score	**Native** **residues**	Beneficial mutant residues	Preservation of function, %
215	9	Valine (V)	Alanine (A), Phenylalanine (F)	100
262	8	Serine (S)	Glycine (G)	88
264	9	Arginine (R)	Histidine (H)	100
288	6	Phenylalanine (F)	Leucine (L)	88
291	8	Glycine (G)	Alanine (A)	100

Note: Residue numbering follows the mature enzyme (starting at 40 of the full‐length sequence). Alignment position begin at position 1 for visualization; therefore, alignment position *n* corresponds to mature residue (*n* + *39*).

### 3.2 Hydrogen Bond Dilution Analysis

**FIGURE 2:**
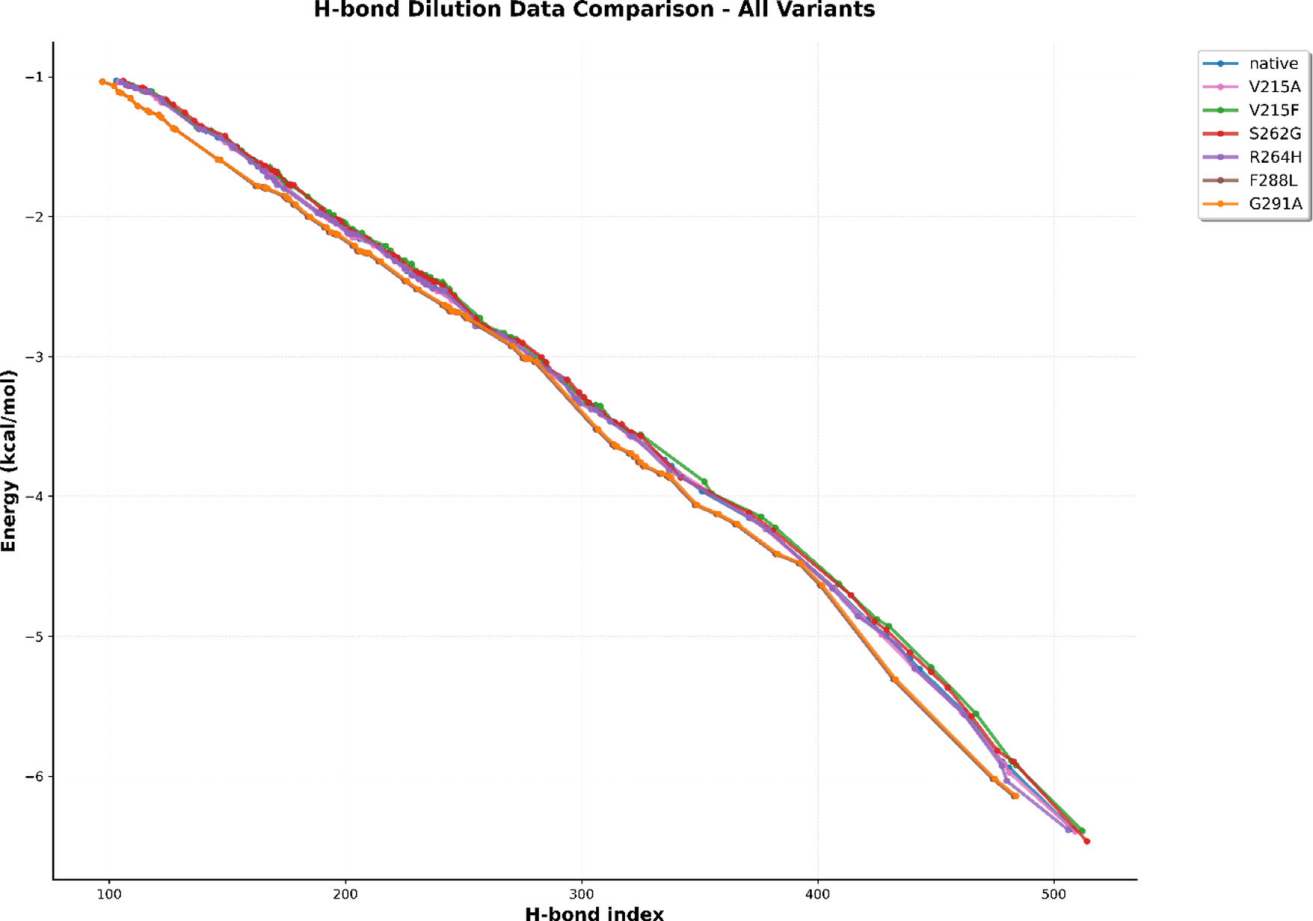
The line graph depicting the simulated thermal denaturation of the enzyme structures.

The conformational flexibility and structural stability of the *B. thuringiensis* chitinase and its six beneficial mutant variants were evaluated through simulated hydrogen bond decomposition analysis. Since noncovalent interactions play a crucial role in maintaining the enzyme's structural integrity, their assessment provides valuable insight into the flexibility and stability of the protein [44]. The resulting hydrogen bond dilution profile reflected the amount of energy (in kcal/mol) required to disrupt all hydrogen bonds within the structure. These findings, therefore, indicated the level of thermal energy necessary to induce structural denaturation of the enzyme. From Figure 2, the native chitinase structure required −6.378 kcal/mol, while the beneficial variants were denatured around −6 kcal/mol with minor variances. Additionally, the observed trajectory for simulated denaturation of the enzyme structure illustrated that the pattern of denaturation for F288L and G291A varied considerably when compared with other beneficial variants, which could potentially be attributed to the conformational changes induced by the point mutations, thus modifying the catalytic activity. Furthermore, to assess the effectiveness of non–covalent interaction properties within the catalytic domain, the beneficial mutant variants, along with the native structure were subjected to molecular docking analysis with the substrate molecule.

### 3.3 Binding Analysis of Beneficial Mutant Variants and Native Enzyme with Chitobiose

**FIGURE 3:**
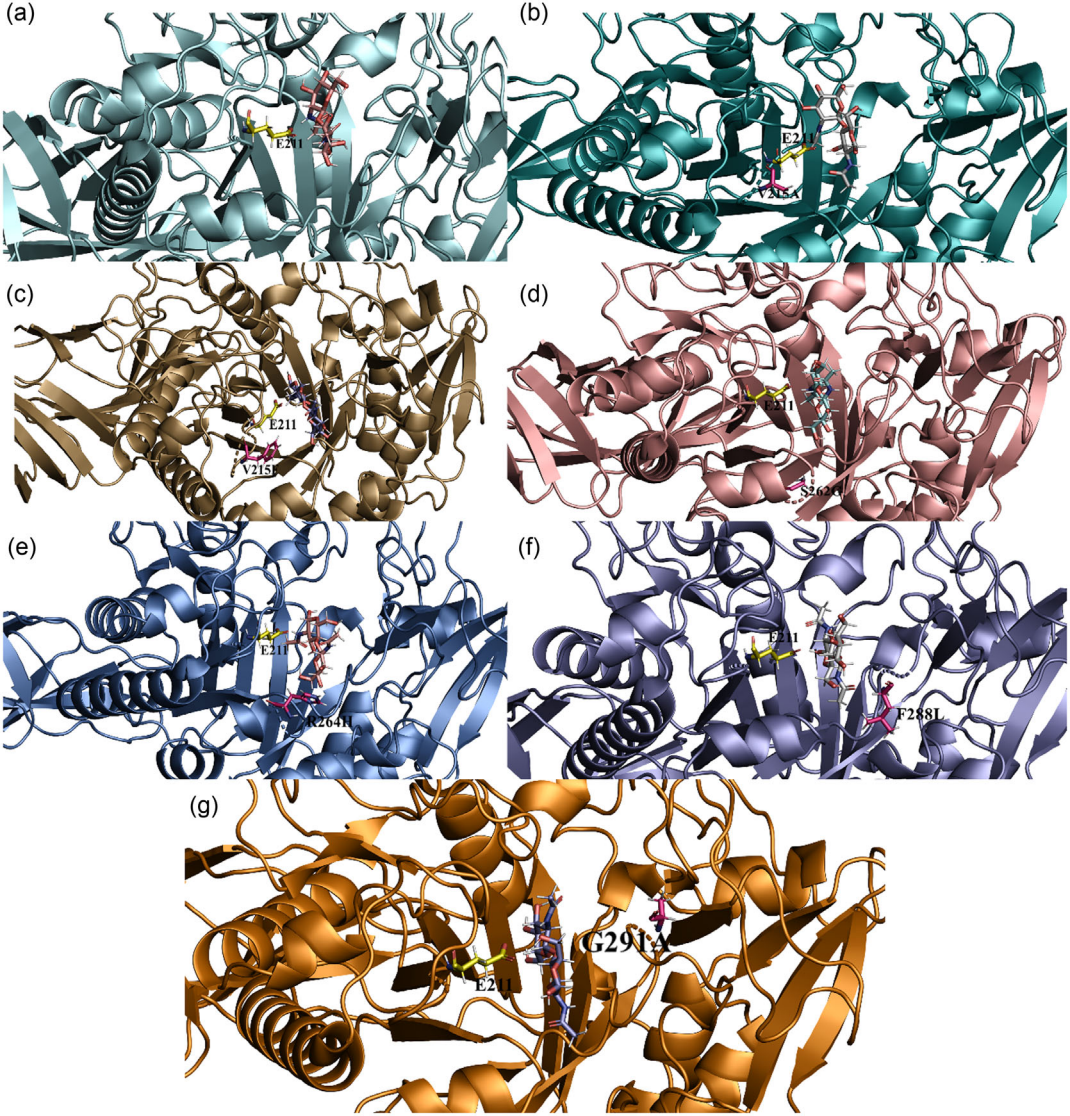
The image illustrating the molecular binding poses focused on the catalytic domain, emphasizing the catalytic residue and the positions of the mutant variant in close proximity to the chitobiose molecule. (a) Native enzyme, (b) V215A, (c) V215F, (d) S262G, (e) R264H, (f) F288L, and (g) G291A.

The molecular interaction demonstrated that the binding free energy of native chitinase with its substrate, chitobiose, was −5.67 kcal/mol, which reflects a strong interaction within its catalytic domain (Figure 3). Besides, the binding free energy analysis of the beneficial mutant variants revealed distinct interaction strengths with the chitobiose. Based on the binding affinity, the beneficial mutant variants exhibited relatively stronger interactions with the substrate compared to the native chitinase. Specifically, the V215A mutant displayed a binding energy of −5.74 kcal/mol, while V215F exhibited −6.88 kcal/mol. Similarly, S262G demonstrated a binding energy of −6.68 kcal/mol, and the R264H variant showed −5.89 kcal/mol. The F288L recorded a binding energy of −5.84 kcal/mol, whereas G291A exhibited −5.98 kcal/mol. These variations in binding energy values highlighted the differential substrate affinities introduced by each mutation. The predicted binding energy of the chitinase structures indicates a strong interaction of chitobiose within the catalytic binding domain, which harbours the conserved active‐site motif sequence “DGVDLDWE,” a crucial determinant of GH18 chitinase activity [45, 46] . This same binding groove and active‐site motif have been consistently observed in several crystal structures of GH18 chitinases. For example, the crystal structure of *Vibrio harveyi* chitinase A complexed with chitooligosaccharides demonstrated that the E315M mutation results in catalytic inactivity [42]. Furthermore, the *B. thuringiensis* ChiA74 structure, utilized in our study, offers a domain‐resolved perspective of a bacterial GH18 chitinase scaffold [27].

Besides, we examined the interactions involving larger chitin subsites by including chitotetraose (four subsites) in the binding analysis with the chitinase enzyme structures (Supplementary Figure S1). The results exhibited a binding affinity pattern consistent with that observed for chitobiose (Supplementary Table S1). However, chitobiose subunits were used in the semiempirical QM calculations to minimize atomic complexity and constraints associated with the semiempirical approach [47, 48]. Moreover, a previous in vitro study by Madhupraksh et al*.* also reported that chitinase D from *Serratia proteamaculans* forms a complex with chitobiose to identify key mutational sites enhancing transglycosylation activity [49]. To further validate this observation, the enzyme–substrate complexes of both the native and mutant chitinases were subjected to SMD simulations.

**FIGURE 4:**
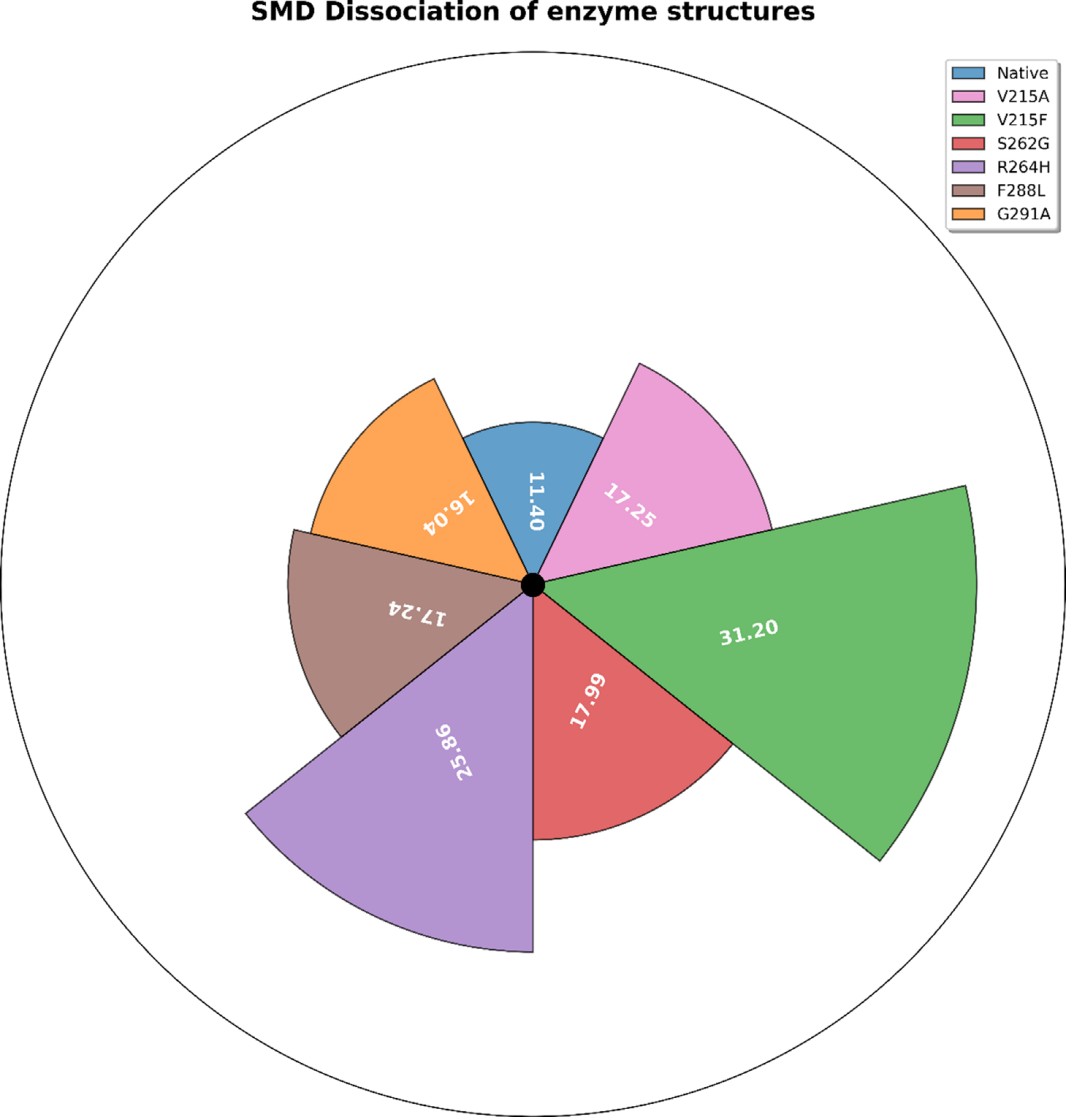
SMD 3D graph defining the time taken by the chitobiose to reach a maximum distance of 30 Å.

The SMD analysis demonstrated the dissociation behaviour of chitobiose from the binding sites of both the native enzyme and the beneficial mutant variants, offering valuable insights into their dynamic binding interactions. A longer dissociation time of the substrate indicates a stronger and more stable binding affinity within the enzyme–substrate complex. As shown in Figure 4, the chitobiose molecule in the native enzyme required 11.40 ps to completely dissociate from its interaction site. In comparison, the substrate dissociation time for the beneficial mutants ranges from 16 to 31 ps, which is relatively longer than those observed for the native chitinase. The figure shows that the V215A and V215F variants took 17.24 and 31.20 ps, respectively, to unbind. Similarly, the dissociation time of chitobiose from the catalytic pockets of the S262G, R264H, F299L, and G291A variants was 17.98, 25.85, 17.24, and 16.04 ps, respectively. These SMD simulations clearly indicated that the beneficial mutants exhibit a significantly stronger binding affinity toward the substrate compared to the native chitinase. To further examine the reactive potential of chitobiose in complex with these enzyme structures, molecular orbital analyses were subsequently performed. Furthermore, SMD simulations were conducted to evaluate the dissociation pattern of the chitotetraose subsites from the chitinase structures (Supplementary Figure S2).

### 3.4 Molecular Orbital Assessment of Chitobiose

Subsequently, the molecular conformation of chitobiose, upon docking was examined to elucidate its reactivity and activity by examining nucleophilicity and electrophilicity, which influence bond formation with the catalytic residue [50]. Elevated nucleophilicity accelerated bond formation and cleavage with the enzyme, while electrophilicity, reflecting the electron‐accepting ability, governs molecular stability and activity [51]. Thus, chitobiose's nucleophilicity and electrophilicity were inferred from its HOMO and LUMO energies, respectively. Results (Table 3) indicated that the HOMO and LUMO energies of chitobiose before docking were −10.10 and −0.90 eV, indicating its strong nucleophilic and electrophilic nature. After docking, the energies of chitobiose bound to native and mutant variants indicated that the HOMO energies remain consistent across all variants, including native enzyme, substantiating its highly reactive characteristic. Furthermore, the native enzyme's chitobiose showed a LUMO energy of 0.98 eV, whereas the beneficial mutants displayed LUMO energies ranging from 1.17 to 0.80 eV. Among these, R264H variant had the lowest value at 0.80 eV, followed by the G291A variant at 0.87 eV. The substrates bound to both of these beneficial variants exhibit lower LUMO energies than those of the native enzyme and the other mutants, indicating that these variants may confer improved substrate binding affinity. To further evaluate the roles of nucleophilicity and electrophilicity in the catalytic reaction, both the native enzyme and the beneficial mutants were subjected to reaction pathway analysis.

**TABLE 3:** The HOMO and LUMO energies along with their energy gap.

Structure before docking	HOMO, eV	LUMO, eV	Energy gap, eV
Chitin (CHT)	−10.10	−0.90	9.2
**Structure after docking**	**HOMO, eV**	**LUMO, eV**	**Energy gap, eV**
Native/CHT	−10.07	0.98	9.09
V215A/CHT	−10.02	1.17	8.85
V215F/CHT	−10.02	1.04	8.98
S262G/CHT	−10.02	1.04	8.98
R264H/CHT	−10.03	0.80	9.23
F288L/CHT	−10.06	1.12	8.94
G291A/CHT	−10.27	0.87	9.4

### 3.5 Simulated Enzyme Kinetics Analysis

**FIGURE 5:**
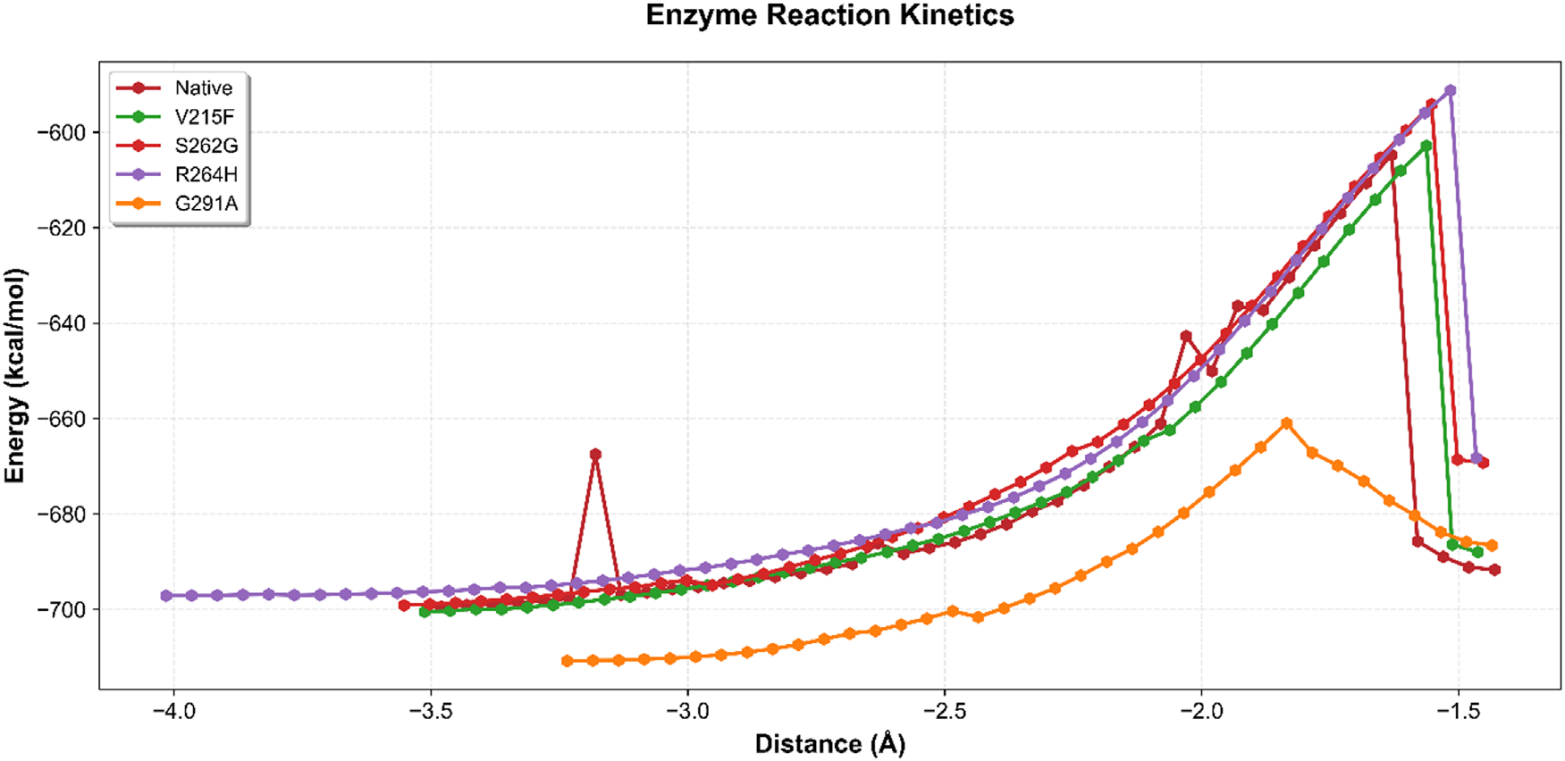
The reaction kinetics graph illustrates the reaction coordinate's path to reach the final formation of the product enthalpy.

To evaluate the enzyme's catalytic properties following mutation, it is essential to determine both its substrate affinity and dynamic conformational changes toward the transition state with minimal activation energy. The predicted reaction activation barrier differences provide insights into the catalytic favourability of modified bacterial chitinase, even though these trends are not directly comparable with the experimental kinetic constants (*k*_cat_ and *k*_m_) but can be considered comparative indicators of transition‐state equilibrium potential. Also, mutations in the binding groove have been reported to enhance the catalytic activity of bacterial chitinase. Previous experimental validations have demonstrated that rationally engineered mutations in bacterial GH18 chitinases can increase activity by more than 60%, significantly enhancing chitinolytic performance [52]. Similarly, site‐directed mutagenesis in *Serratia marcescens* chitinase has been reported to improve catalytic activity and enhance thermophilic properties [40]. Accordingly, the reaction coordinate pathway (Figure 5) depicted the molecular trajectory of the reactant as it reaches the transition state and completes the reaction enthalpy formation. The activation energies of the native enzyme and the beneficial mutant variants were analysed within this framework. The native chitinase activation energy was computed to be 94.66 kcal/mol. For the beneficial mutant variants, V215F and F288L, the conformational reaction coordinates failed to attain the self‐consistent field, which impacted the convergence of the reaction mechanism [53]. The activation energies for V215A, S262G, R264H, and G291A were found to be 97.66, 105.04, 105.95, and 49.83 kcal/mol, respectively. Among the beneficial variants, G291A exhibited the lowest activation energy, identifying it as the most beneficial mutant compared to both the native enzyme and the other mutants.

The G291A mutant demonstrated both catalytic favourability and structural stability through improved substrate accommodation and reduced activation energy, while retaining a stability and flexibility profile comparable to that of the native bacterial chitinase, as confirmed by hydrogen bond dilution and rigidity analyses. Moreover, the observed decrease in activation energy correlates with enhanced substrate affinity, as evidenced by docking, SMD, and molecular orbital analyses. This dual‐functional behaviour highlights G291A's potential for achieving efficient product conversion in large‐scale catalytic applications.

## 4 Conclusion

Chitinases play a pivotal role in the eco‐friendly conversion of chitin derived from seashell waste. To sustain effective bioconversion under challenging conditions, these enzymes can be strategically improved through in silico site‐directed mutagenesis. In this study, the catalytic domain of *B. thuringiensis* chitinase was used as a structural model to computationally identify beneficial mutations. Comprehensive interaction analyses demonstrated that the engineered mutants exhibited substantially enhanced substrate affinity relative to the native enzyme, representing a critical advancement toward optimized enzyme performance. Moreover, molecular orbital analysis revealed that substrate binding to these mutant variants, especially the G291A variant, exhibited enhanced nucleophilic and electrophilic characteristics. These electronic enhancements suggested a more favourable environment for catalytic bond formation and stabilization of transition states. Overall, the G291A variant emerges as a promising candidate for biotechnological applications, including bioseparation and large‐scale chitin conversion. The insights gained from this mutation offer a valuable template for designing beneficial modifications in related enzyme systems.

## Supporting Information

Additional supporting information can be found online in the Supporting Information section. **Supporting** **Fig. S1**: The image illustrates the molecular binding poses focused on the catalytic domain, emphasising the catalytic residue and the mutant variant in proximity to the chitotetraose molecule. a) Native enzyme, b) V215A, c) V215F, d) S262G, e) R264H, f) F288L, g) G291A. **Supporting Fig. S2**: SMD 3D graph defining the time taken by the chitotetraose to reach a maximum distance of 30 Å. **Supporting** **Table S1**: The table values shows the comparative binding scores between chitotetraose and chitobiose when docked with chitinase structures

## Author Contributions

SGSA conceived and wrote the manuscript. GC, ASV, AS, and PK contributed to the computational studies. RR designed and supervised the research. GC and RR reviewed and edited the final manuscript. All authors provided feedback on earlier drafts. Everyone read and approved the final version.

## Declaration of Generative AI and AI‐assisted Technologies in the Writing Process

During the preparation of this work, the authors utilised ProWritingAid to improve content clarity. After employing this tool, the authors reviewed and edited the content as necessary and took full responsibility for the research article's content.

## Conflicts of Interest

The authors declare no conflicts of interest.

## Supporting information

Supplementary Material
