# Supplementary material for: Computer‐Based Design to Improve Bacillus thuringiensis Chitinase for Industrial Applications
Source: ChemistryOpen. 2026 Apr 1;15(4):e70190. doi: 10.1002/open.70190 (PMC13045421; doi:10.1002/open.70190)
Supplement: Supplementary file 1 — Supplementary Material [file OPEN-15-e70190-s001.pdf]

Supplementary Information

1. Molecular Docking analysis of beneficial mutants and native enzyme with chitotetraose

To check the interaction between larger subunits of chitin, the chitotetraose (four subsites) was involved in the binding analysis with the chitinase enzyme structures (supplementary Figure 1), which indeed followed the same binding pattern as chitobiose (supplementary table 1).

Supplementary Table 1. The table values shows the comparative binding scores between chitotetraose and chitobiose when docked with chitinase structures

**Sl.no**	**Chitinase (CHN) Structures**	**CHN/Chitotetraose**	**CHN/Chitobiose**
1	Native	-8.0 kcal/mol	-5.67 kcal/mol
2	V215A	-8.0 kcal/mol	-5.74 kcal/mol
3	V215F	-8.6 kcal/mol	-6.88 kcal/mol
4	S262G	-8.1 kcal/mol	-6.68 kcal/mol
5	R264H	-8.0 kcal/mol	-5.89 kcal/mol
6	F288L	-8.3 kcal/mol	-5.84 kcal/mol
7	G291A	-8.0 kcal/mol	-5.98 kcal/mol

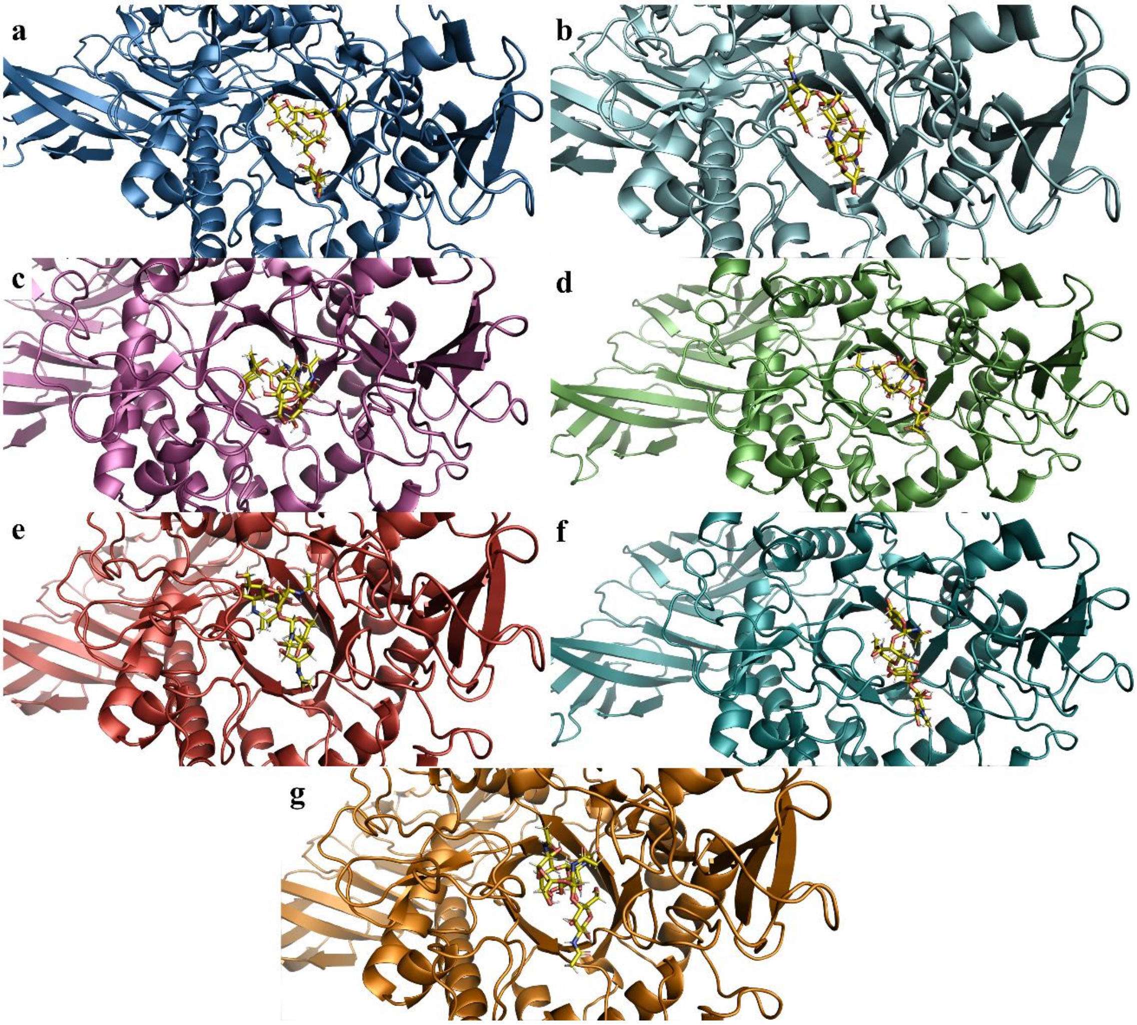

Supplementary Figure 1: The image illustrates the molecular binding poses focused on the catalytic domain, emphasising the catalytic residue and the mutant variant in proximity to the chitotetraose molecule. a) Native enzyme, b) V215A, c) V215F, d) S262G, e) R264H, f) F288L, g) G291A

2. Steered Molecular Dynamics simulations analysis of beneficial mutants and native enzyme with chitotetraose

SMD was evaluated to analyse the disassociation pattern of chitotetraose from the docked complexes of chitinase structures. Supplementary Figure 2 illustrates that the chitotetraose took 17.31 ps to unbind from the native chitinase structure, while the unbinding time of chitotetraose from G291A took 24.64 ps. This prediction supports the claim that the chitotetraose holds greater interactions with the catalytic binding groove of the G291A beneficial mutant.

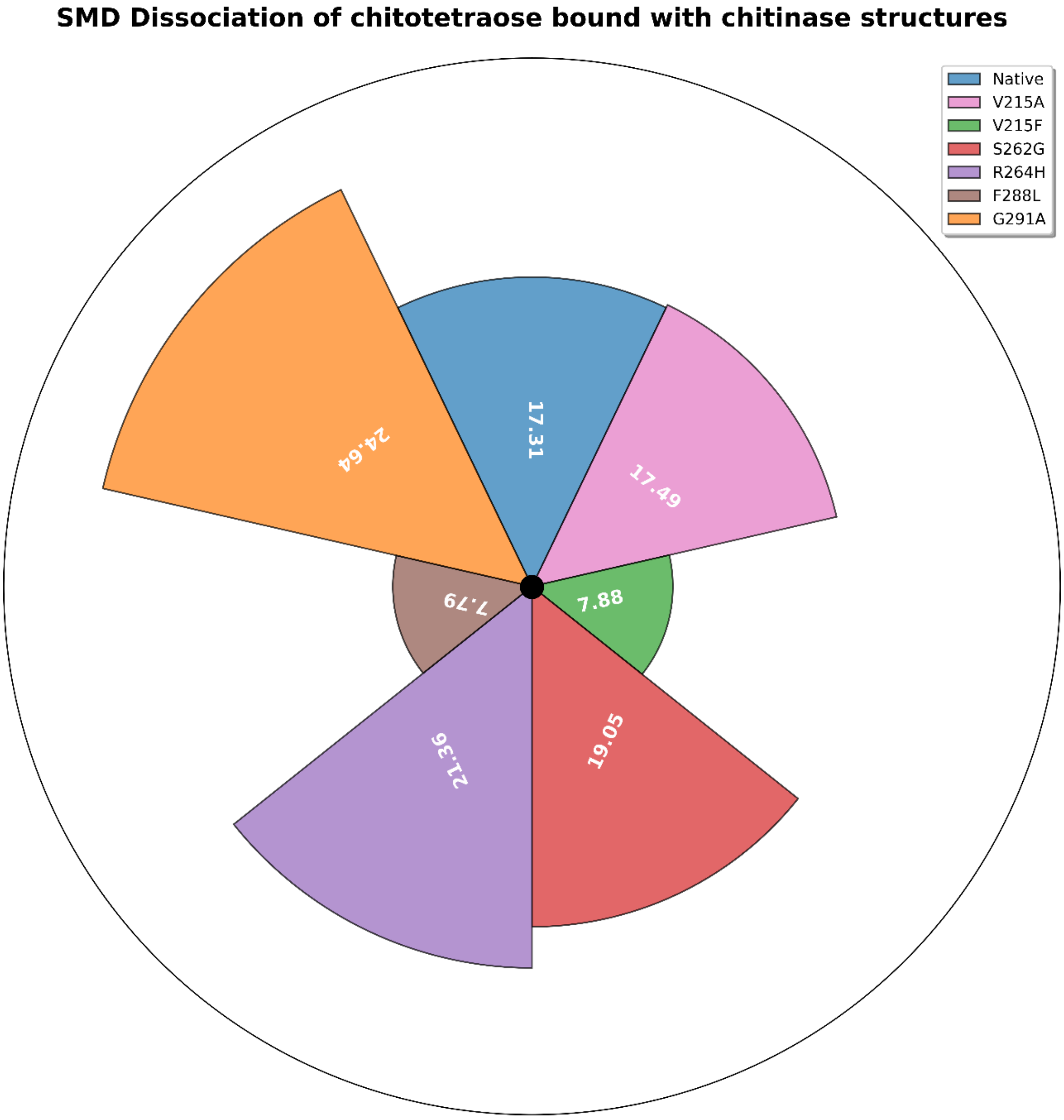

Supplementary Figure 2: SMD 3D graph defining the time taken by the chitotetraose to reach a maximum distance of 30 Å
